# NF-κB Inducing Kinase, a Central Signaling Component of the Non-Canonical Pathway of NF-κB, Contributes to Ovarian Cancer Progression

**DOI:** 10.1371/journal.pone.0088347

**Published:** 2014-02-12

**Authors:** Masaya Uno, Yasunori Saitoh, Kanako Mochida, Eri Tsuruyama, Tohru Kiyono, Issei Imoto, Johji Inazawa, Yasuhito Yuasa, Toshiro Kubota, Shoji Yamaoka

**Affiliations:** 1 Department of Comprehensive Reproductive Medicine, Tokyo Medical and Dental University, Tokyo, Japan; 2 Department of Molecular Virology, Tokyo Medical and Dental University, Tokyo, Japan; 3 Department of Molecular Oncology, Graduate School of Medicine, Tokyo Medical and Dental University, Tokyo, Japan; 4 Department of Molecular Cytogenetics, Medical Research Institute and School of Biomedical Science, Tokyo Medical and Dental University, Tokyo, Japan; 5 Virology Division, National Cancer Center Research Institute, Tokyo, Japan; 6 Department of Human Genetics, Institute of Health Biosciences, The University of Tokushima Graduate School, Tokushima, Japan; University of Navarra, Spain

## Abstract

Ovarian cancer is one of the leading causes of female death and the development of novel therapeutic approaches is urgently required. Nuclear factor-κB (NF-κB) is constitutively activated in several types of cancer including ovarian cancer and is known to support the survival of cancer cells. However, molecular mechanisms of persistent activation of NF-κB in ovarian cancer remain largely unknown. We report here that, in addition to the previously reported canonical activation, NF-κB is activated through the noncanonical pathway in ovarian cancer cells. RNA interference-mediated silencing of NF-κB inducing kinase (NIK), a central regulator of the noncanonical pathway, reduced the NF-κB2/p52 DNA binding activity and NF-κB-dependent reporter gene expression as well as NF-κB target gene expression. Notably, anchorage-dependent and -independent cell growth was impaired in NIK-depleted cells. Depletion of NIK also suppressed tumor formation in the nude mouse xenograft assay. These results indicate that NIK plays a key role in constitutive NF-κB activation and the progression of ovarian cancer cells and suggest that NIK represents an attractive therapeutic target for ovarian cancer.

## Introduction

Epithelial ovarian cancer is one of the most lethal gynecological malignancies and its survival rate is much lower than other cancers that affect women. Since the ovarian cancer initially exhibits subtle and nonspecific symptoms, most of the patients present with advanced disease so that aggressive surgical treatment in combination with chemotherapy remains the standard of care. Although advances in chemotherapy improved survival of ovarian cancer patients, they often do not respond to initial chemotherapy or relapse after achieving a favorable response [1]. Therefore, new therapeutic approaches are urgently required to achieve better treatment outcome.

NF-κB is a transcription factor involved in diverse biological processes such as immune response, inflammation, cancer and cell death [2]. In mammalian cells, NF-κB is composed of homo- and heterodimers of five members, NF-κB1 (p50 and its precursor p105), NF-κB2 (p52 and its precursor p100), RelA (p65), RelB and c-Rel. In resting cells, the activity of NF-κB is tightly regulated by its interaction with inhibitory IκB proteins. The precursor protein p105 undergoes constitutive processing by the cellular proteasome that eliminates the IκB-like C-terminal region to generate p50. In contrast, p52 production requires IκB kinase (IKK)-induced phosphorylation and proteasome-mediated processing of p100. NF-κB signaling is mediated by two pathways called the canonical and noncanonical pathways. Activation of the canonical pathway is mainly triggered by cytokine stimuli such as tumor necrosis factor-α (TNFα) and interleukin-1β, followed by activation of the IKK complex, which consists of two protein kinases IKKα and IKKβ and a regulatory protein NF-κB essential modulator (NEMO also named IKKγ). Activated IKK-induced phosphorylation of IκBα leads to its polyubiquitination and proteasomal degradation, followed by translocation of the p50-RelA heterodimer to the nucleus and induction of target gene expression. The noncanonical NF-κB pathway is activated through particular TNF receptor family members such as B cell-activating factor (BAFF) receptor, CD40 and lymphotoxin beta receptor that bind to the TNF receptor-associated factor (TRAF) 2 or TRAF3. Noncanonical NF-κB activation has been reported to rely on elevated expression of NF-κB-inducing kinase (NIK), which is achieved in two ways either by impairment of K48 polyubiquitination of NIK or by enhanced mRNA expression. In unstimulated cells, TRAF3 links NIK to a multi-subunit E3 ubiquitin ligase complex composed of TRAF2 and cellular inhibitor of apoptosis 1 and 2 (cIAP1 and cIAP2), leading to K48 polyubiquitination and proteasomal degradation of NIK, thus maintaining NIK expression at a low level. In response to stimulation with cytokines such as BAFF or CD40 ligand, TRAF3 is recruited to the receptor and undergoes ubiquitination-mediated proteasomal degradation. This results in stabilization and accumulation of newly synthesized NIK, while these stimuli do not increase the *NIK* mRNA level [3], [4]. In hematopoietic cancer cells such as multiple myeloma and adult T-cell leukemia as well as lung cancer cells, either stabilization of the NIK protein through impaired negative regulation by the TRAF3/TRAF2/cIAP complex or aberrant expression of the *NIK* mRNA have been reported [5], [6], [7], [8]. In any case, accumulation of NIK results in activation of the IKK complex, which in turn phosphorylates p100 leading to its processing to p52 and nuclear translocation of the p52/RelB heterodimer. In contrast to the activation of the canonical pathway, noncanonical NF-κB activation does not require association of NEMO with the IKK complex and is relatively persistent [9].

Previous reports showed constitutive activation of NF-κB and its contribution to the manifestation of malignant phenotype in several types of cancer. NF-κB activation results in elevated expression of genes related to cell cycle progression, survival and invasion of cancer cells. For example, overexpression of cyclin D1, an important regulator of the cell cycle, promotes cancer cell proliferation, while deregulated expression of B-cell lymphoma-xl protects cancer cells from apoptosis. In addition, matrix metallopeptidase 9 (MMP-9) and vascular endothelial growth factor promotes tumor invasion and angiogenesis [10]. As for ovarian cancer, inhibition of IKKβ activity, either by a small molecule kinase inhibitor or by RNAi-mediated gene silencing, was reported to suppress proliferation and invasion of ovarian cancer cell lines [11]. Blockade of NF-κB signaling by expression of a dominant negative form of IκBα altered tumorigenesis of ovarian cancer cell lines [12]. In addition, accumulation of nuclear RelA in ovarian tumors was reported to associate with poor prognosis [13]. Nevertheless, the mechanisms underlying the persistent NF-κB activation in ovarian cancer cells have remained largely unknown. Rattan et al. showed that the expression of transcription elongation factor A-like 7, a suppressor of RelA-dependent gene transcription, is frequently down-regulated in ovarian cancer cells [14]. We recently reported elevated expression of NIK and its role in oncogenic properties of adult T-cell leukemia and lung cancer cells, in which *NIK* mRNA was aberrantly expressed [7], [8]. In the present study, we demonstrate important roles for NIK in the proliferation *in vitro* and tumorigenicity of ovarian cancer cells.

## Materials and Methods

### Ethics Statement

Experiments using primary ovarian cancer samples were approved by the ethical committee of Tokyo Medical and Dental University and written informed consents were obtained from all patients. All animal experiments were performed with the approval of the Animal Study Committee of Tokyo Medical and Dental University (Permit No. 0120286A) and conformed to all relevant guidelines and laws.

### Cell culture and primary samples

Four human ovarian cancer cell lines, RMG-I, RMUG-S, RMUG-L and MCAS were obtained from the Japanese Collection of Research Bioresources Cell Bank (Tokyo, Japan) and 2 ovarian cancer cell lines, OMC-3 and JHOC-5, were from the RIKEN Cell Bank of Japan (Tsukuba, Japan) [15]. RMG-I, RMUG-S, RMUG-L and OMC-3 were cultured in Ham's F-12 medium supplemented with 10% FBS. JHOC-5 was cultured in 1∶1 mixture of Dulbecco's modified Eagle's medium (DMEM) and Ham's F12, containing 0.1 mM non-essential amino acids supplemented with 10% fetal bovine serum (FBS). MCAS was cultured in Eagle's Minimum Essential Medium containing 20% FBS. All of the other ovarian cancer cell lines were described elsewhere [16], [17], [18]. Human embryonic kidney cell lines, HEK293 and HEK293T were cultured in DMEM containing 10% FBS. HOSE1C is an immortalized human ovarian surface epithelial cell line established from primary human ovarian surface epithelium (HOSE) cells following infection with retroviruses expressing mutant Cdk4, cyclinD1 and human telomerase reverse transcriptase [19]. HOSE1C cells were cultured in 1∶1 mixture of DMEM and Ham's F12 supplemented with 10% FBS. All media used were supplemented with 100 U/mL of penicillin G and 100 µg/mL of streptomycin sulfate. Primary ovarian cancer samples were obtained from 12 patients in an affiliated hospital of the Tokyo Medical and Dental University. The median age of patients was 56.5 years, ranging from 27 to 80 years. Of the 12 ovarian cancers, 5 were histologically serous carcinoma, 4 were clear cell carcinoma, 2 were endometrioid carcinoma and 1 was yalk sac tumor.

### Preparation of cell extracts

For preparation of whole-cell extracts, cells were harvested and lysed in RIPA buffer [20 mM tris(hydroxymethyl)aminomethane-HCl (pH 7.5), 137 mM NaCl, 1% Nonidet P-40 (NP-40), 0.5% deoxycholate, 0.1% SDS]. For extraction of cytoplasmic and nuclear proteins, cells were firstly lysed in hypotonic buffer [20 mM 4-(2-hydroxyethyl)-1-piperazineethanesulfonic acid (HEPES) (pH 7.8), 0.15 mM ethylenediaminetetraacetic acid (EDTA), 0.15 mM ethyleneglycoltetracetic acid, 10 mM KCl] and incubated on ice for 10 minutes. NP-40 was added to a final concentration of 1%, and the cell suspensions were centrifuged at 14,000 rpm for 5 minutes. The supernatants were used as cytoplasmic extracts. The pellets were washed three times with isotonic buffer [20 mM HEPES (pH 7.8), 100 mM NaCl, 0.1 mM EDTA and 25% glycerol] and resuspended in nuclear extraction buffer [20 mM HEPES (pH 7.8), 400 mM NaCl, 0.1 mM EDTA, 25% glycerol and 1 mM dithiothreitol (DTT)]. After 30 minutes of incubation at 4°C with constant agitation, the suspension was centrifuged at 14,000 rpm for 2 minutes. The supernatants were recovered and used as nuclear extracts. The RIPA, hypotonic and nuclear extraction buffers were supplemented with 1 µg/mL aprotinin, 1 µg/mL leupeptin, 0.57 mM phenylmethanesulfonylfluoride, 100 µM sodium vanadate, and 20 mM β-glycerophosphate. The protein concentration was determined by the Bradford assay.

### Electrophoretic mobility shift assay (EMSA)

Five µg of nuclear extracts were incubated for 2 hours at room temperature in binding buffer [10 mM HEPES (pH 7.8), 100 mM NaCl, 1 mM EDTA, 1 mM DTT, 2.5% glycerol and 0.5 µg of poly (dI-dC)] with 0.5 ng of ^32^P-labeled NF-κB-specific probe derived from the H-2Kb promotor or ^32^P-labeled Oct-1 probe [20], [21]. For super-shift assays, nuclear extracts were preincubated with specific antibodies or antiserum for 1 hour on ice before incubation with the labeled probe. The following antibodies or antiserum were used for the preincubation: antibody to p50 (Santa Cruz Biotechnology, Santa Cruz, CA, USA, #sc-7178 X), purified rabbit IgG (Cedarlane Laboratories, Hornby, Canada) and anti-p52 serum (Upstate Biotechnology, Lake Placid, NY, USA, 06-413). Pre-immune serum, anti-RelA and anti-RelB antisera were kindly provided by Drs. N.R. Rice (NCI, MA) and A. Israël (Institut Pasteur, Paris). For competitive binding assay, nuclear proteins were incubated with 100-fold molar excess cold oligonucleotide before adding the labeled probe. The protein-DNA complexes were separated on a polyacrylamide gel containing 2.5% glycerol followed by autoradiography.

### NF-κB DNA-binding activity

The binding of p52 or RelB to NF-κB binding sequence was quantified with TransAM™ NF-κB Family Kit (Active Motif, Carlsbad, CA, USA) according to the manufacturer's instructions. Briefly, 5 µg of nuclear extracts were added to a 96-well plate pre-coated with the oligonucleotide containing NF-κB consensus sequence. The activated p52 or RelB present in the extracts binding to this nucleotide was detected by secondary antibodies conjugated to HRP.

### Immunoblotting

Whole-cell (30 µg), cytoplasmic (30 µg) and nuclear (10 µg) lysates were resolved by SDS-PAGE and analyzed by immunoblotting. The following antibodies were used: anti-NF-κB2 p52 (C-5) (Santa Cruz Biotechnology, #sc-7386) for detection of p52 and its precursor p100; anti-NIK (Cell Signaling, Danvers, MA, USA, #4994); anti-RelB (C-19) (Santa Cruz Biotechnology, #sc-226); anti-phospho-p100 (Ser866/870) (Cell Signaling, #4810); anti-phospho-IκBα (Ser32/36) (5A5) (Cell Signaling, #5205); anti-IκBα (C-21) (Santa Cruz Biotechnology, #sc-371); anti-phospho-IKKα(Ser180)/IKKβ (Ser181) (Cell Signaling, #2681), anti-IKKα (H-744, Santa Cruz Biotechnology, #sc-7218), anti-IKKα/β (H-470, Santa Cruz Biotechnology, #sc-7607), anti-TRAF2 (C90-481) (BD Biosciences, San Jose, CA, USA, 558890); anti-TRAF3 (H-122) (Santa Cruz Biotechnology, SC-1828); anti-cIAP1 (R&D systems, Minneapolis, MN, USA, AF8181); anti-lamin A/C (4C11) (Cell signaling, #4774); anti-α-tubulin (Sigma-Aldrich, St Louis, MO, USA, T9026). For detection of endogenous NIK protein, cells were treated with 0.1% DMSO or 20 µM of MG132 (PEPTIDE INSTITUTE, Osaka, Japan) for 6 hours and cytoplasmic extracts were subjected to immunoblot analysis.

### Real-time reverse transcription-PCR (RT-PCR) analysis

Real-time RT-PCR analysis was performed essentially as described previously [7]. Briefly, total RNA was extracted from cell lines and ovarian cancer tissues using ISOGEN (Nippon Gene, Tokyo, Japan) according to the manufacturer's instructions. The mRNA levels of *NIK*, *Cyclin D1* and *MMP-9* were quantified using StepOnePlus real-time RT-PCR system (Applied Biosystems, Foster City, CA, USA). One hundred ng of total RNA was subjected to quantitative RT-PCR using TaqMan® One-Step RT-PCR Master Mix Reagents Kit (Applied Biosystems). Reverse transcription was performed at 48°C for 30 minutes and Taq DNA polymerase was activated at 95°C for 10 minutes, followed by 45 amplification cycles of denaturing at 95°C for 15 seconds and annealing and extension at 60°C for 1 minute. The mRNA levels were normalized to the 18S ribosomal RNA levels measured by real-time RT-PCR using 100 pg of total RNA. Expression of miR-31 was examined using TaqMan® Micro RNA Assays (Applied Biosystems) according to manufacturer's instructions. The miR-31 levels were normalized with the RNU48 expression levels.

### Lentiviruses

Lentiviral vectors capable of expressing shRNA targeting *NIK* (pCS-puro-NIKi-1 and pCS-puro-NIKi-2) were described previously [7]. The targeting sequence specific for *green fluorescent protein* (GFP) 5′-ACCCGCGCCGAGGTGAAG-3′ was inserted immediately downstream of the H1 promoter of the pSuperRetoro vector (Oligoengine, Seattle, WA, USA), generating pSR-GFPi. The shRNA expression cassette was transferred to lentiviral vector, pCS-puro-PRE, carrying the puromycin resistance gene expressed under the control of the phosphoglycerate kinase promoter [7]. The resultant plasmid was named pCS-puro-GFPi. For production of lentiviruses capable of expressing shRNA, HEK293T cells were cotransfected with pCS-puro-GFPi (shCtl), pCS-puro-NIKi-1 (shNIK-1) or pCS-puro-NIKi-2 (shNIK-2) together with the pCMV-ΔR8.2 packaging construct and pHCMV-VSV-G (kind gifts from Dr. I.S.Y. Chen) using FuGENE6 (Promega, Madison, WI, USA). Culture supernatants were collected and filtered 56 hours after transfection. RMG-I and JHOC-5 cells were infected with these lentiviruses for 12 hours in the presence of 10 µg/mL polybrene. Infected cells were selected in the presence of 4 µg/mL puromycin (Sigma-Aldrich) 24 hours after infection.

### NF-κB reporter gene assay

RMG-I and JHOC-5 cells were infected with lentivirus vectors carrying either a NF-κB-responsive promoter-driven Firefly *luciferase* gene (CS-κB-R2.2) or the elongation factor (EF)-1α promoter-driven *Renilla luciferase* gene (pCERp) [8], [22]. Resultant cell pools were subsequently infected with lentiviruses capable of expressing each shRNA. After selection with 4 µg/mL puromycin for 3 days, cells were harvested and luciferase activities were measured using Dual-Luciferase Reporter Assay System (Promega). NF-κB-dependent Firefly luciferase activity was normalized with EF-1α promoter-driven dependent *Renilla* luciferase activity.

### Cell-cycle analysis

Cells were fixed in 70% ethanol and incubated with 0.5 mg/mL RNase for 45 minutes and then stained with 30 µg/mL propidium iodide for 30 minutes at room temperature. The DNA content of cells was measured using FACS Calibur system (BD Biosciences) and analyzed CellQuest software (BD Biosciences).

### Soft agar assay

RMG-I cells expressing shCtl or shNIK-1 were seeded in F-12 medium containing 0.33% agar. After 4 weeks of incubation, colonies larger than 60 µm in diameter were counted to evaluate anchorage-independent cell growth.

### Mouse xenograft tumor model

BALB/cAJcl-nu/nu mice (CLEA Japan, Tokyo, Japan) were maintained under specific pathogen-free condition at the Experimental Animal Center of Tokyo Medical and Dental University. RMG-I cells expressing shCtl or shNIK-1 were suspended in serum free medium. Athymic mice (5–6 weeks old) were inoculated subcutaneously in the postauricular region with 5×10^6^ cells. Tumor volume was recorded every week. The greatest longitudinal diameter (length) and the greatest transverse diameter (width) were measured with a caliper. Tumor volume was calculated using the following formula: tumor volume = length×width^2^/2. All mice were sacrificed 3 weeks after inoculation and the weight of each tumor was measured.

### Statistics

Data are presented as mean ± SD or as representative images of at least three independent experiments. The statistical analysis was performed using the two-tailed Student's t-test. For the patient sample analysis and the mouse model, significant differences between groups were determined by the Mann-Whitney U test. A *P* value of <0.05 was considered statistically significant.

## Results

### Noncanonical NF-κB activation in ovarian cancer cells

**Figure 1:**
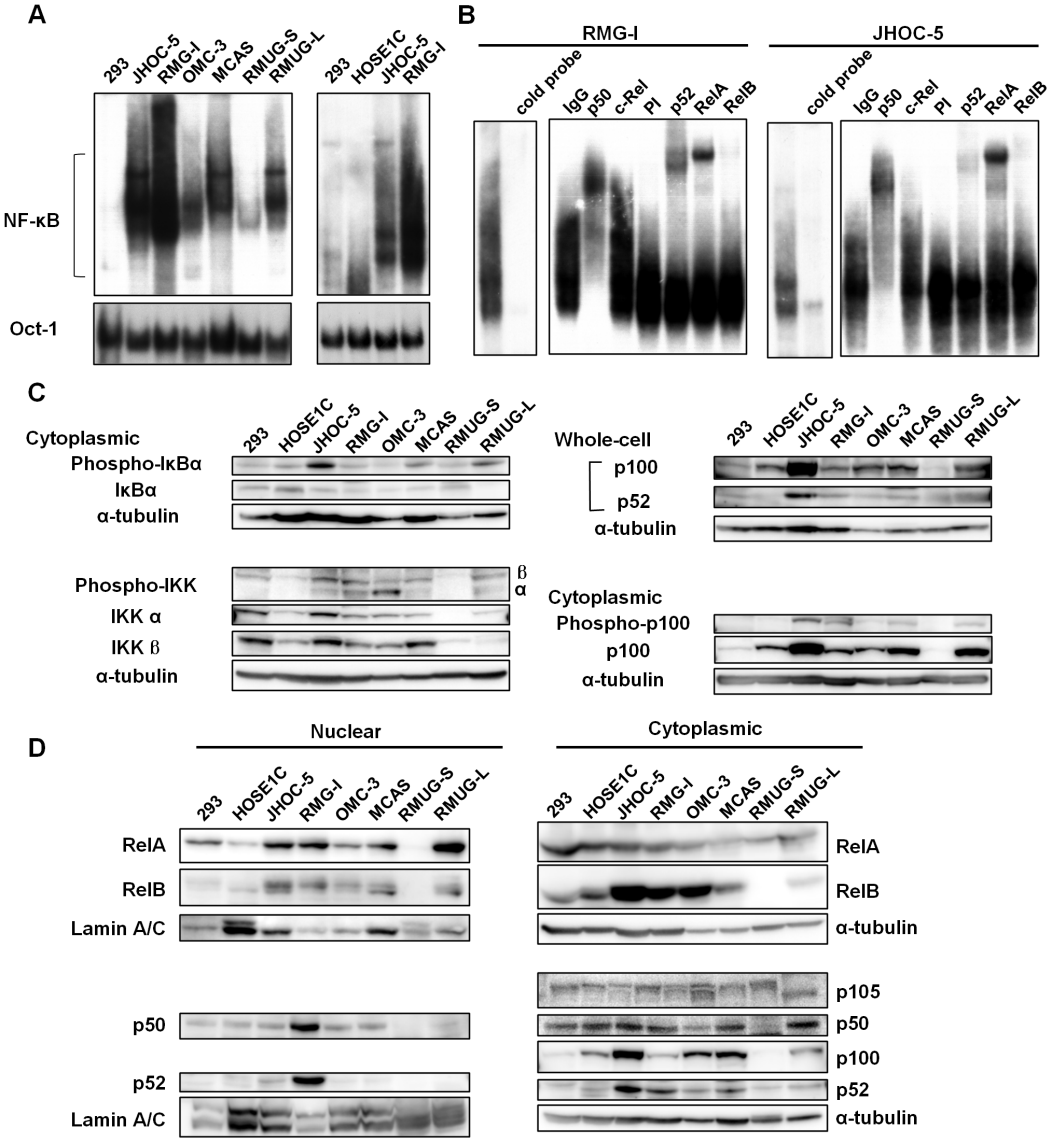
Constitutive activation of NF-κB in ovarian cancer cell lines. (A) Five micrograms of nuclear extracts from the indicated cell lines were subjected to EMSA using ^32^P-labeled oligonucleotides containing an NF-κB binding sequence or Oct-1-binding sequence as probes. (B) For competition assay nuclear samples were preincubated with 100-fold molar excess cold oligonucleotide before adding the labeled probe (left panel). DNA-binding NF-κB components in the indicated cell lines were determined by super-shift EMSA (right panel). Nuclear extracts (5 µg) from the indicated cell lines were preincubated for 30 minutes with purified mouse IgG, anti-p50 or anti-c-Rel antibodies, pre-immune (PI), anti-p52, anti-RelA or anti-RelB sera, and then subjected to EMSA with the NF-κB-specific probe. (C and D) Nuclear (10 µg), cytoplasmic (30 µg) or whole-cell (30 µg) extracts were subjected to SDS-PAGE followed by immunoblottings with the indicated antibodies.

To explore how NF-κB is constitutively activated in ovarian cancer cells, we firstly examined NF-κB DNA binding activity by EMSA. Ovarian cancer cells showed markedly elevated NF-κB DNA-binding activity compared to HEK293 cells known to have a basal NF-κB activity. HOSE1C, an immortalized ovarian surface epithelial cell line showed an NF-κB binding activity as low as HEK293 cells and was used as a control in the following studies (Figure 1A). The binding to the radio-labeled probe was efficiently competed with an excess amount of cold probe, demonstrating the specificity of the binding (Figure 1B). Even though the persistent activation of NF-κB has been reported in ovarian cancer, NF-κB-DNA binding complexes involved in these cells have remained unknown. Super-shift assays revealed that not only NFKB1/p50 and RelA, but also NFKB2/p52 and RelB were involved in the NF-κB DNA-binding complexes in RMG-I and JHOC-5 cells (Figure 1B). Immunoblottings showed these cell lines expressed a specifically phosphorylated forms of IKKα, IKKβ and IκBα (Figure 1C). Phosphorylation of IKKβ and IκBα was more intense in JHOC-5 and MCAS cells while IKKα was predominantly phosphorylated in RMG-I and OMC-3 cells, suggesting preferential activation of the canonical and noncanonical pathways in each cell lines. The fact that expression of NFKB2/p100 largely depends on NF-κB activation through the canonical pathway may partly explain the abundant expression of NFKB2/p100 and its phosphorylated form as well as p52 in JHOC-5 cells. The phosphorylated form of p100 was detected in the ovarian cancer cell lines except for RMUG-S, but not in control 293 or HOSE1C cells. The presence of RelA and RelB in the nucleus correlated with the results of EMSA (Figure 1D). These results indicate that both the canonical and/or noncanoncal NF-κB pathways are differentially activated in ovarian cancer cells.

### NIK plays a pivotal role in constitutive NF-κB activation in ovarian cancer cells

**Figure 2:**
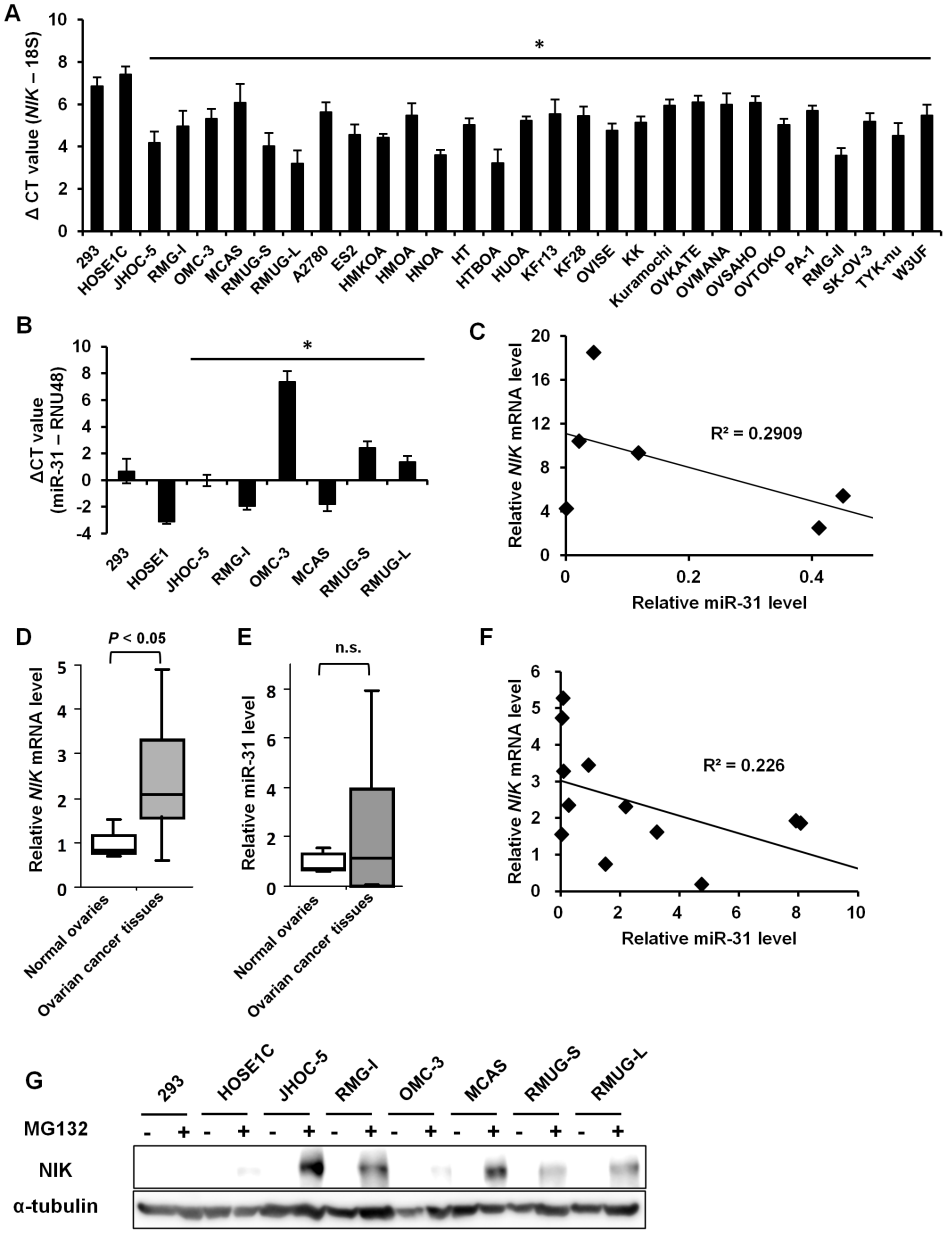
NIK is aberrantly expressed at the pretranslational level in ovarian cancer cells. (A and D) Total RNA was extracted from the indicated cell lines or ovarian cancer tissues and subjected to real-time RT-PCR for quantifying *NIK* mRNA expression. (A) The ΔCT is determined as the difference between the Ct value of *NIK* mRNA and that of diluted 18S rRNA. Single asterisks denote significant decrease (*P*<0.05) in the ΔCT value compared to HOSE1C cells. (D) The *NIK* mRNA levels were normalized to 18S RNA. Relative *NIK* mRNA levels are shown by fold increase in the mRNA abundance relative to the average of normal ovaries (arbitrarily set at 1). (B and E) Total RNA used in the panels A and D was subjected to real-time RT-PCR for miR-31 quantification. (B) The ΔCT is determined as the difference between the Ct value of miR-31 and that of internal control RNU48. Single asterisks denote significant increase (*P*<0.05) in the ΔCT value compared to HOSE1C cells. (E) The miR-31 levels were normalized to the corresponding RNU48 levels. Relative miR-31 levels are shown by fold increase in the miR-31 abundance relative to the average of normal ovaries (arbitrarily set at 1). (C, F) Pearson correlation coefficient R^2^ between the NIK mRNA and the miR-31 levels was calculated. (G) Thirty micrograms of lysates were subjected to SDS-PAGE followed by immunoblottings with the indicated antibodies. For detection of endogenous NIK, cells were treated with MG132 (20 µM) or DMSO for 6 hours before preparation of cytoplasmic extracts. n.s., not significant.

Elevated expression of NFKB2/p52 prompted us to examine NIK expression in ovarian cancer cells. Quantitative RT-PCR analysis revealed that control HOSE1C cells showed larger CT difference than the 28 ovarian cancer cell lines tested, among which 17 cell lines expressed 4- to 16-fold more *NIK* mRNA than control HOSE1C cells (Figure 2A). A recent report demonstrated that loss of microRNA miR-31 targeting *NIK* underlies constitutive activation of NF-κB in adult T-cell leukemia cells [23]. We therefore evaluated miR-31 expression in ovarian cancer cells by quantitative RT-PCR. Four out of 6 cancer cell lines had less than 1/8 of miR-31 RNA in HOSE1C cells although correlation between *NIK* and miR-31 RNA expression was not significant (Figure 2B and 2C). Importantly, quantitative RT-PCR analysis of ovarian cancer tissues revealed a median of 2.4-fold increase in *NIK* mRNA expression compared to normal ovarian tissues (Figure 2D). However, miR-31 expression in ovarian cancer tissues did not significantly differ from those in normal ovarian tissues nor statistically correlated with *NIK* mRNA expression (Figure 2E and 2F). Thus, certain cases of *NIK* mRNA accumulation cannot solely be explained by reduced miR-31 expression and suggest previously unveiled mechanism(s) of *NIK* mRNA overexpression.

Expression of the NIK protein is tightly regulated by polyubiquitination and proteasome-mediated degradation and is generally kept undetectable by immunoblotting without inhibiting its rapid degradation. The expression of TRAF2, 3 and cIAP1 in ovarian cancer cell lines that negatively regulate NIK expression remained comparable to control cells (Figure S1). As we were unable to detect the NIK protein by simple immunoblotting or immunoprecipitation-coupled immunoblotting using cytoplasmic lysates from these cell, we treated cells with a proteasome inhibitor MG132 prior to protein extraction. The NIK protein was readily detectable in ovarian cancer cells in the presence of MG132, but not in control HOSE1C nor HEK293 cells (Figure 2G), indicating enhanced production of the NIK protein in ovarian cancer cells. These data collectively indicate that NIK is aberrantly expressed at the pretranslational level in ovarian cancer cells.

**Figure 3:**
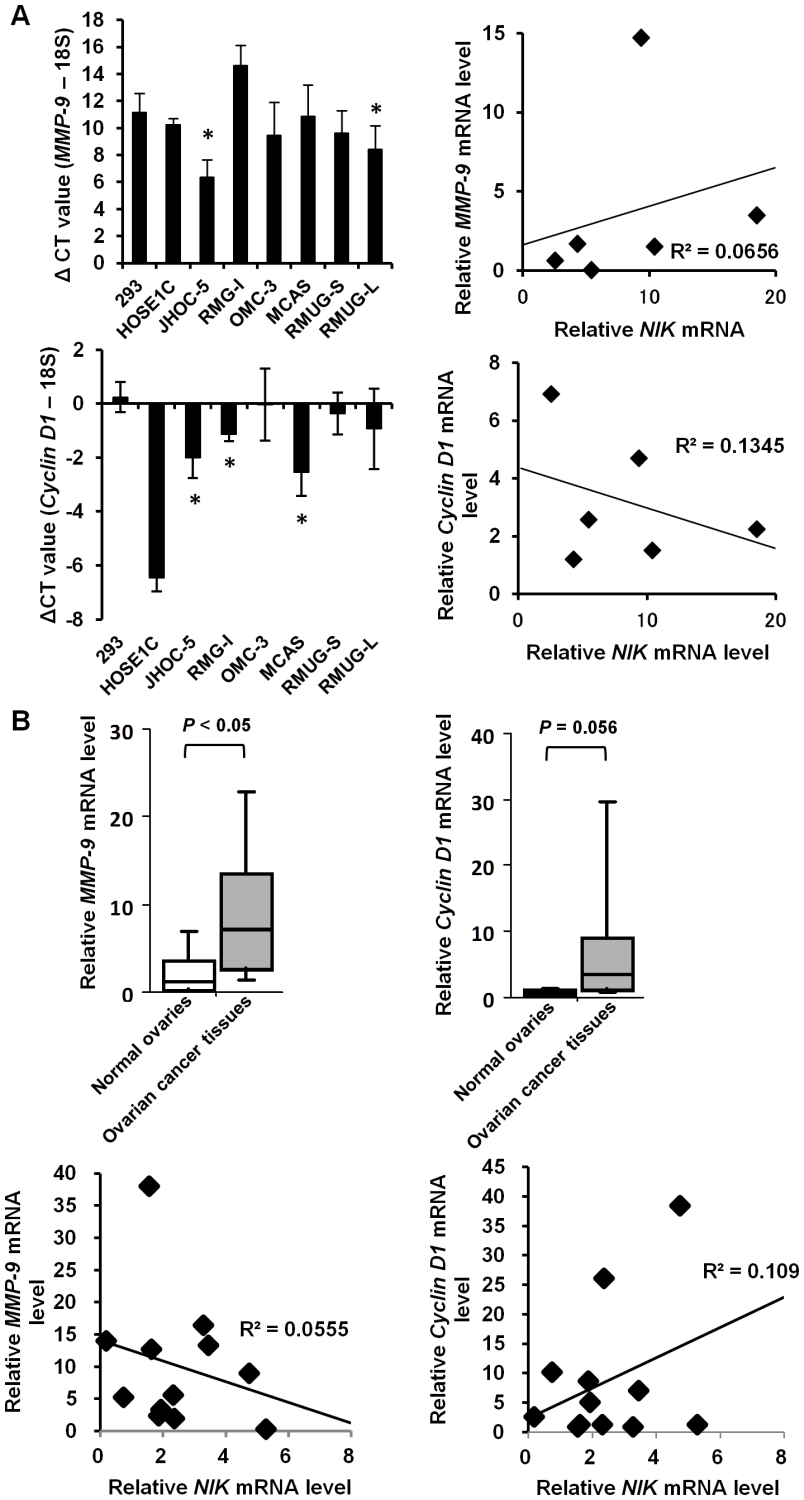
*MMP-9* and *Cyclin D1* mRNAs are highly expressed in ovarian cancer cells. Total RNA extracted from the indicated cell lines (A) or ovarian cancer tissues (B) was subjected to real-time RT-PCR for quantifying *MMP-9* or *Cyclin D1* mRNA expression. (A) The ΔCT is determined as the difference between the Ct value of *MMP-9* or *Cyclin D1* mRNA and that of diluted 18S rRNA. Single asterisks denote significant difference (*P*<0.05) in the ΔCT value compared to HOSE1C cells (*MMP-9*) or HEK293 cells (*Cyclin D1*). (B) The mRNA levels were normalized to 18S RNA. Relative mRNA levels are shown by fold increase in the mRNA abundance relative to the average of normal ovaries (arbitrarily set at 1). Pearson correlation coefficient R^2^ between *MMP-9* or *Cyclin D1* and *NIK* mRNA levels was shown. Relative mRNA levels are calculated by fold increase in the mRNA abundance relative to that of HOSE1C cells (*MMP-9*) or HEK293 cells (*Cyclin D1*).

Previous reports showed that poor prognosis of ovarian cancer patients correlates with overexpression of pro-MMP-9 and cyclin D1 [24], [25], whose expression is known to be regulated by NF-κB. Quantitative RT-PCR analyses demonstrated that *MMP-9* mRNA levels of most of the ovarian cancer cell lines were higher than that of control HOSE1C cells. The *MMP-9* mRNA levels in each cell line did not correlate with the *NIK* mRNA levels (Figure 3A). Levels of *cyclinD1* mRNA in all the tested ovarian cancer cell lines exceeded that of HEK293 cells, while HOSE1C cells abundantly expressed *cyclinD1* mRNA due to forced expression of this gene for immortalization. In ovarian cancer tissues, *MMP-9* mRNA expression was significantly up-regulated and *Cyclin D1* mRNA levels tended to increase in ovarian cancer tissues although these mRNA expression in each sample did not correlate with *NIK* mRNA expression (Figure 3B).

**Figure 4:**
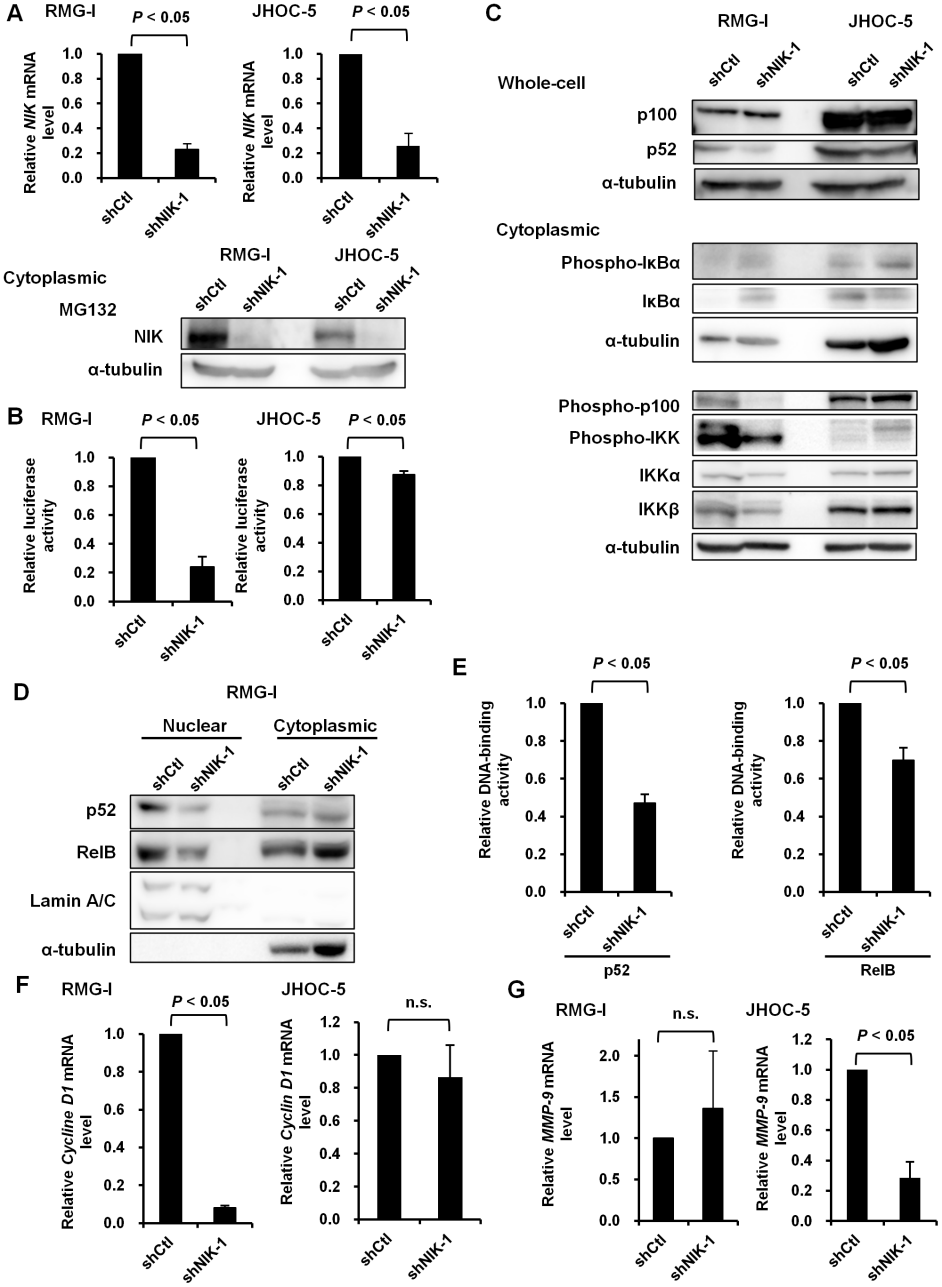
NIK is responsible for noncanonical NF-κB activation in ovarian cancer cell lines. (A) RMG-I and JHOC-5 cells were infected with lentivirus vectors expressing shRNA targeting *NIK* (shNIK-1) or *GFP* (shCtl). Total RNA was extracted from these cells and subjected to real-time RT-PCR for quantifying *NIK* mRNA expression. The *NIK* mRNA levels were normalized to 18S RNA. Relative *NIK* mRNA levels are shown by fold increase in the mRNA abundance relative to the average of shCtl (arbitrarily set at 1). Single asterisks denote significant decrease (*P*<0.05) compared to shCtl cells. In parallel, cells were treated with MG132 (20 µM) for 6 hours before preparation of cytoplasmic extracts. Cytoplasmic extracts (30 µg) were subjected to immunoblot analysis with the indicated antibodies. (B) RMG-I and JHOC-5 cells infected with a lentivirus vector carrying an NF-κB-dependent Firefly luciferase expression cassette and that carrying an EF-1α promoter-dependent *Renilla* luciferase expression cassette. Established cell pools were infected with lentivirus vectors expressing shRNA targeting *NIK* (shNIK-1) or *GFP* (shCtl). The cells were selected with puromycin (4 µg/mL) for 72 hours and subjected to the dual luciferase assay, in which Firefly luciferase activity was normalized with *Renilla* luciferase activity. Relative luciferase activities are expressed as light unit compared to the control (shCtl). (C) Thirty micrograms of whole-cell or cytoplasmic extracts from the cells shown in (A) were subjected to immunoblot analysis with the indicated antibodies. (D) Nuclear (10 µg) or cytoplasmic (30 µg) extracts were subjected to immunoblot analysis with the indicated antibodies. (E) Five micrograms of nuclear extracts was assayed for measuring NF-κB DNA-binding activity. Relative p52 and RelB DNA binding activities are shown by fold change relative to those of shCtl expressing cells. (F, G) Expression of *MMP-9* (F) and *Cyclin D1* (G) was analyzed by real-time RT-PCR using total RNA extracted from RMG-I or JHOC-5 cells expressing shRNA targeting *NIK* (shNIK-1) or *GFP* (shCtl). Relative mRNA levels are shown as fold change compared to the control (shCtl). n.s., not significant.

To explore how NIK regulates NF-κB-dependent gene transcription in ovarian cancer cell lines, we established an NF-κB reporter cell system by lentivirus transduction. RMG-I and JHOC-5 cells were infected with lentivirus vectors carrying a Firefly *luciferase* transcription unit under the control of NF-κB-dependent promoter or *renilla luciferase* transcription unit under the control of constitutive elongation factor 1α-derived promoter as an internal control. Established cell pools were subsequently infected with lentivirus capable of expressing either shRNA targeting *NIK* or *GFP* and subjected to luciferase assays. Depletion of NIK was verified by quantitative RT-PCR and immunoblotting in the presence of MG132 (Figure 4A). NIK depletion reduced p52 expression and NF-κB-dependent reporter gene expression in RMG-I and JHOC-5 cells (Figure 4B and 4C). Expression of another shRNA targeting *NIK* mRNA resulted in similar suppression of NF-κB-dependent transcription, excluding the possibility of off target effects (Figure S2A, B).

Previous studies showed that NIK activates the canonical NF-κB pathway as well as the noncanonical pathway [7], [8]. To investigate the role for NIK in canonical and noncanonical NF-κB activation in ovarian cancer cells, we examined the expressions of NF-κB signal transduction molecules in NIK-depleted RMG-I and JHOC-5 cells (Figure 4C). Silencing of NIK caused significant decrease in p52, the specifically phosphorylated forms of IKKα and p100 and p52 in RMG-I, while these effects were only marginal in JHOC-5 cells. Phoshorylation of IκBα was rather increased (Figure 4C). The enhanced IκBα phosphorylation may potentially be a result of compensation by the canonical pathway.

Moreover, NIK depletion reduced nuclear expression of p52 and RelB (Figure 4D). These results are consistent with the enzyme-linked immunosorbent assay (ELISA)-based assay showing that NIK silencing suppressed the DNA-binding activity of noncanonical NF-κB components, p52 and RelB in RMG-I cells (Figure 4E). These results suggest that activation of NIK is critically involved in noncanonical NF-κB activation in these ovarian cancer cell lines.

As shown in Figure 1B and 1C, JHOC-5 cells showed less p52 DNA binding and stronger IκBα phosphorylation compared with RMG-I cells, suggesting predominant canonical NF-κB activation in JHOC-5 cells. Although NIK is supposed to primarily regulate the noncanonical NF-κB pathway in ovarian cancer cells, the predominant canonical NF-κB activation may partly explain the limited reduction in NF-κB-dependent reporter gene expression in NIK-depleted JHOC-5 cells. As expected, reduction of the *Cyclin D1* mRNA expression, which mainly depends on the noncanonical pathway, was evident in NIK-depleted RMG-I cells (Figure 4F). NIK depletion also suppressed *MMP-9* mRNA expression in JHOC-5 cells (Figure 4G).

### NIK regulates anchorage-dependent and -independent growth of ovarian cancer cells

**Figure 5:**
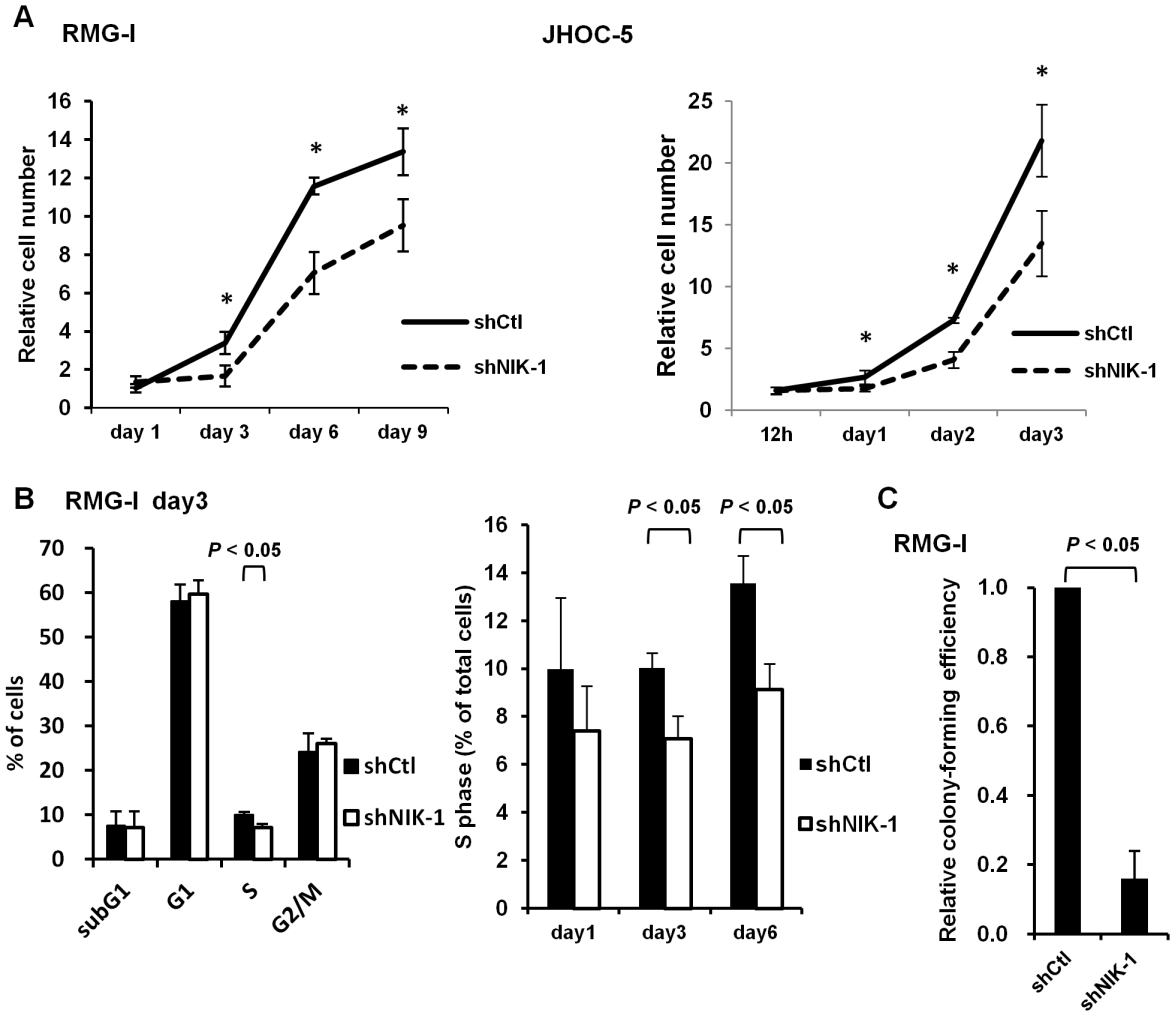
NIK contributes to anchorage-dependent and -independent cell growth of ovarian cancer cell lines. (A) Proliferation of RMG-I and JHOC-5 cells expressing shRNA targeting *NIK* (shNIK-1) or *GFP* (shCtl) was assessed by the trypan blue exclusion test. Relative cell number is determined as fold increase in cell number relative to the number of cells initially plated is shown. Single asterisks denote significant difference (*P*<0.05) between shNIK-1 and shCtl. (B) NIK-depleted RMG-I cells shown in Figure 4 were cultured for additional 3 days and examined for DNA content on each day by flow cytometric analysis with propidium iodide staining. Percentage of cells in each phase is shown (left panel). Percentage of cells in the S phase on day 1, 3 and 6 is shown (right panel). (C) NIK-depleted RMG-I cells shown in Figure 4 were cultured in soft agar medium for three weeks. More than 20 fields at ×40 magnification were randomly chosen and colonies larger than 60 µm were counted. Relative colony forming efficiency is shown as fold change compared to the control (shCtl). n.s., not significant.

We next sought to determine if NIK contributes to the ovarian cancer cell growth. Silencing of NIK resulted in significant reduction of RMG-I and JHOC-5 cell growth on monolayer (Figure 5A). Long-time cell culture did not give a marked difference in cell number because control cells partly reached the confluency. Depletion of NIK with another shRNA also decreased the growth of RMG-I cells (Figure S2C). To investigate whether NIK depletion was cytotoxic or cytostatic, cell cycle distribution was assessed by flow cytometric analysis. NIK depletion decreased the proportion of RMG-I cells in the S phase, but did not increase those in the subG1 phase (Figure 5B and data not shown). Moreover, NIK silencing did not increase the proportion of annexin V-positive RMG-I cells (Figure S3). These results indicate that NIK depletion in RMG-I cells triggered proliferation arrest rather than cell death on monolayer. We also examined if depletion of NIK affects anchorage-independent cell growth by soft-agar colony formation assay. NIK depletion in RMG-I cells decreased colony-forming efficiency without significantly altering the average size of colonies (Figure 5C and Figure S2D). Overall, these results indicate that NIK plays an important role in anchorage-dependent and -independent ovarian cancer cell growth.

### NIK supports tumorigenicity of RMG-I cells

**Figure 6:**
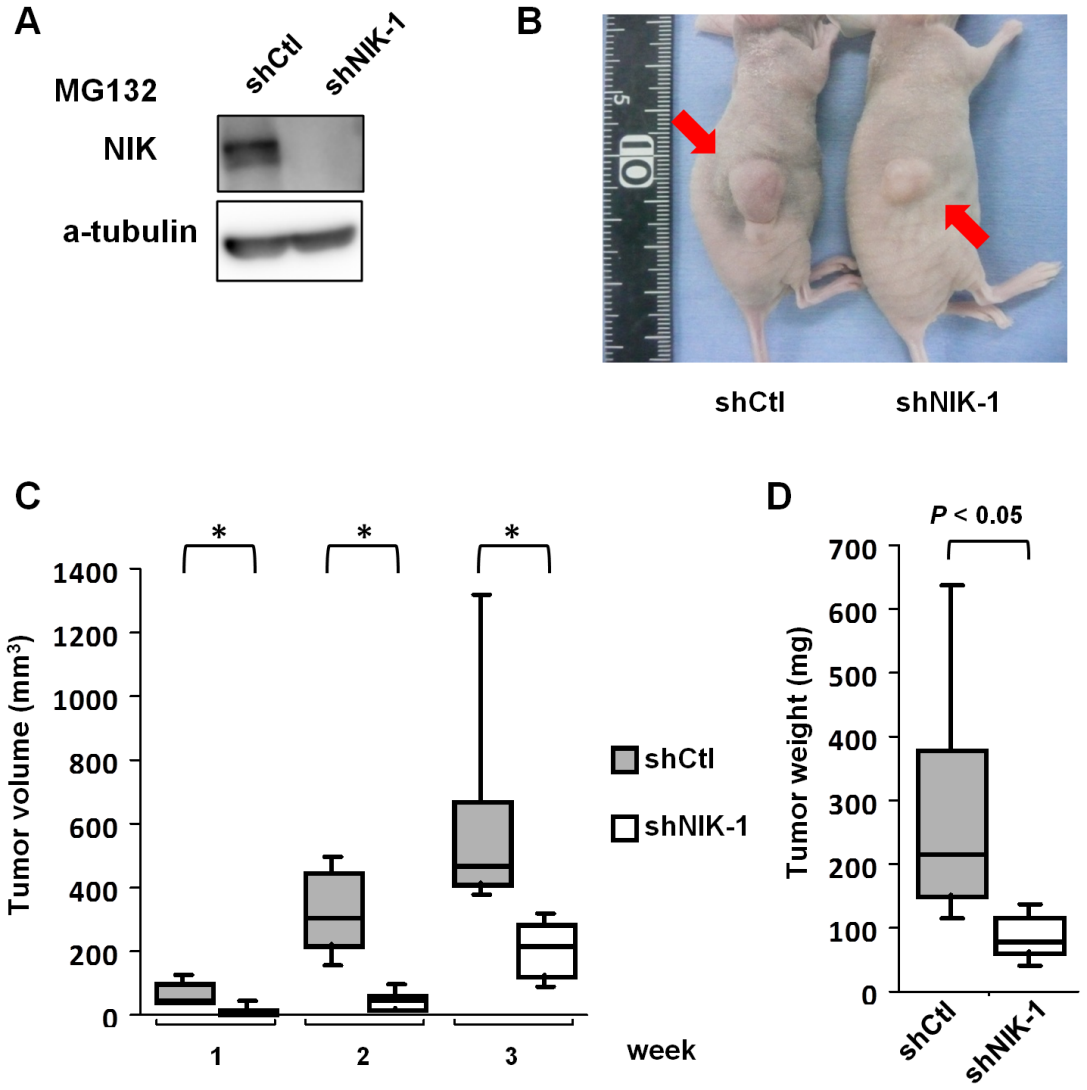
Depletion of NIK in RMG-I cells impaired tumorigenicity. RMG-I cells were infected with lentiviral vectors capable of expressing shRNA targeting *NIK* (shNIK-1) or *GFP* (shCtl) and selected with puromycin for 3 days. Female BALB/cAJcl-nu/nu mice (8 mice/group) were inoculated subcutaneously with these cells (5×10^6^ cells per mouse). (A) Cells used for inoculation were treated in parallel with MG132 (20 µM) for 6 hours and examined for NIK protein expression by immunoblot analysis. (B) A representative photograph of nude mice at 3 weeks after cell inoculation. Red arrows indicate the xenograft tumor. (C) Tumor volume was determined by weekly caliper measurement. Single asterisks denote significant difference (*P*<0.05) between cells expressing shNIK-1 and shCtl. (D) Three weeks after inoculation, tumors were removed and the tumor weight was measured.

We finally examined if NIK contributes to the tumorigenicity of ovarian cancer cells. RMG-I cells were previously reported to be tumorigenic in a mouse xenograft model [26]. As shown in Figure 6A, NIK-depleted or control RMG-I cells were subcutaneously xenografted in nude mice and allowed to grow for 3 weeks. NIK depletion in RMG-I cells apparently delayed tumor formation (Figure 6B); NIK depletion resulted in 2.7-fold reduction in tumor weight and 2.1-fold that in tumor volume compared to control cells (Figure 6C and D). These results indicate that NIK supports the *in vivo* growth of RMG-I cells.

## Discussion

NF-κB activation has been considered to play important roles in the oncogenic properties of cancer cells, while certain studies reported anti-oncogenic roles of canonical NF-κB activation in ovarian cancer cells. Blockage of the canonical pathway by treatment with an IκBα phosphorylation inhibitor was reported to inhibit paclitaxel-induced apoptosis of ovarian cancer cells [27]. Yang et al. showed that canonical NF-κB activation exerts its pro-apoptotic or anti-apoptotic effect in parental or chemoresistant variant of ovarian cancer cell lines, respectively [28]. Thus, inhibition of the canonical pathway would need a cautious approach for treatment of ovarian cancer. On the other hand, roles for activation of the noncanonical pathway in ovarian cancer cells have not extensively been studied. Several lines of evidence indicate important roles for activation of the noncanonical NF-κB pathway in the manifestation of malignant phenotype of cancer cells. Depletion of RelB was reported to alter the viability of Hodgkin lymphoma cell lines and suppress the tumorigenicity of prostate cancer cells [29], [30]. In this study, we show for the first time NIK contributes to the cell growth and tumorigenicity of ovarian cancer cells.

In fact, previous reports indicate that noncanonical NF-κB activation regulates gene expression involved in cancer cell proliferation and invasion. NF-κB2/p52 and RelB are known to bind to NF-κB binding sites in the *Cyclin D1* promoter in TNF-stimulated mammary epithelial cells [31]. Fritz et al. showed that scaffold protein connector enhancer of KSR 1 induces MMP-9 and membrane-type 1 MMP expressions via activation of the noncanonical NF-κB pathway [32]. In addition, transgenic overexpression of the NF-κB p100/p52 subunit in mammary epithelium using the β-lactoglobulin milk protein promoter was reported to increase the expression levels of cyclin D1, cyclo-oxygenase-2, MMP-2 and MMP-9 [33]. With ovarian cancer cells, however, little has been known about how noncanonical NF-κB activation contributes to expression of these genes. In line with previous reports, this study demonstrated that NIK supports the expression of the *Cyclin D1* mRNA in RMG-I cells and *MMP-9* mRNA in JHOC-5 cells, respectively.

Several studies showed that loss-of-function mutations of TRAF2, TRAF3 or cIAP1/2 protects NIK from proteasomal degradation leading to accumulation of NIK and subsequent constitutive noncanonical NF-κB activation in multiple myeloma cells [5], [6]. A recent report also demonstrated a mechanism of deregulated NIK activity showing that a fusion oncoprotein of apoptosis inhibitor 2 and mucosa associated lymphoid tissue lymphoma translocation gene 1 cleaves NIK at R325 resulting in production of NIK lacking the TRAF3-binding site, which enables constitutive noncanonical NF-κB signaling in MALT lymphoma [34]. In our present study, the NIK protein was barely detectable without treatment of ovarian cancer cells with the proteasome inhibitor MG132, indicating a rapid turnover of the NIK protein in those cells. Accordingly, the expression of proteins involved in K48 polyubiquitination-mediated degradation of NIK, such as TRAF2, TRAF3 and cIAP1 was readily detectable. These results suggest that the cause of noncanonical NF-κB activation in ovarian cancer cells cannot simply be attributed to dysregulation of NIK degradation. In our study, JHOC-5 and MCAS cells exhibited NF-κB activation through the both pathways as revealed by phosphorylation of IKKβ and IκBα. While NIK is known to trigger the both pathways, the reporter assay results in NIK-depleted JHOC-5 cells suggest limited contribution of NIK to constitutive NF-κB activity in these cells (Figure 4 B). Nevertheless, results shown in Figures 5 and 6 indicate that NIK plays important roles in the cell growth and tumorigenicity.

Our quantitative RT-PCR analysis revealed enhanced NIK expression at the pretranslational level in ovarian cancer cells. Several mechanisms of *NIK* mRNA overexpression have been considered. Demethylation and acetylation of histone H3 was reported to be involved in over-expression of the *NIK* mRNA in breast cancer cells [35]. Previous reports showed that gene amplification of *nik* resulted in elevated *NIK* mRNA expression in myeloma and lung cancer cells [5], [8]. In addition, miR-31 was reported to negatively regulate NIK expression in ATL and melanoma cells [23], [36]. Wyman et al., using deep sequencing of small RNA cDNA libraries, showed that miR-31 expression was under-expressed among ovarian cancer tissues compared to normal ovarian surface epithelium cultures [37]. We showed impaired expression of miR-31 in 5 out of 12 ovarian cancer tissues and *NIK* mRNA expression was found to be elevated in 4 out of those 5 cases. These observations raise a possibility that down-regulation of miR-31 expression underlies NIK overexpression in some but not all ovarian cancer cells.

Targeting constitutive NF-κB activation has been considered as an effective strategy for cancer therapy. Bortezomib is a well-known compound as a proteasome inhibitor inducing cancer cell death and is the first NF-κB inhibitor that got approval to enter clinical trials in ovarian cancer treatment. In preclinical studies, bortezomib showed significant anti-tumor activity, but a phase 2 study revealed that it had minimal activity as a single-agent treatment for recurrent ovarian cancer [38], [39]. Since inhibition of proteasomal degradation affects a wide variety of physiological and pathological processes, it is supposed to influence the viability of normal cells as well as cancer cells. Thus, in the development of potent anti-cancer agents, targeting a specific mechanism supporting cancer cell proliferation should provide a better way to establish an effective treatment strategy. A recent work revealed that a small molecule NIK inhibitor, 4H-isoquinoline-1,3-dione, which specifically targets ATP-binding site of NIK, reduced the viability of Hodgkin lymphoma cell lines [29]. Although characterization of *aly* mice and *nik* knockout mice suggested that NIK is required for lymphorganogenesis [40], [41], no information has been available of if NIK inhibition affects the health of adult individuals. Obviously, more work will be needed to clarify that NIK inhibition represents a safe and effective way of ovarian cancer treatment.

## Supporting Information

Figure S1 **Thirty micrograms of cytoplasmic extracts were subjected to SDS-PAGE followed by immunoblotting with the anti-TRAF2, anti-TRAF3 or anti-cIAP1 antibodies.**
(TIF)

Figure S2 (**A**) RMG-I cells were infected with lentiviral vectors capable of expressing shRNA targeting *NIK* (shNIK-2) or *GFP* (shCtl) followed by selection with puromycin (4 µg/mL) for 72 hours. These cells were treated with 20 µM of MG132 for 6 hours and cytoplasmic extracts (30 µg) were subjected to SDS-PAGE and immunoblottings using anti-NIK or anti-α-tubulin antibodies. Expression of *NIK* mRNA was analyzed by real-time RT-PCR using total RNAs extracted these cells. (**B**) RMG-I cells transduced with lentiviruses carrying an NF-κB-dependent Firefly *luciferase* expression cassette and an EF-1α promoter-dependent *Renilla luciferase* expression cassette were infected with lentivirus vectors capable of expressing shRNA targeting *NIK* (shNIK-2) or *GFP* (Ctli). These cells were selected with puromycin (4 µg/mL) for 72 hours and subjected to the dual luciferase assay, in which Firefly luciferase activity was normalized by *Renilla* luciferase activity. Relative luciferase activities are expressed as light unit compared to the control (shCtl). (**C**) proliferation of NIK-depleted RMG-I cells in panel A was assessed by trypan blue exclusion test. Relative cell numbers is expressed as fold change compared to the number of cells plated. Single asterisks denote significant difference (*P*<0.05) between cells expressing shNIK-2 or shCtl. (**D**) NIK-depleted RMG-I cells in panel A were cultured in soft agar medium for three weeks and then cell colonies larger than 60 µm in diameter were counted under more than 20 microscopic fields at 40× magnification. Relative colony-forming efficiency is expressed as fold change compared to the control (shCtl).
(TIF)

Figure S3 **NIK-depleted RMG-I cells shown in** **Figure 5B** **were stained with FITC-conjugated Annexin-V and analyzed by flow cytometry.**
(TIF)

Methods S1 **Apoptosis assay.**
(DOC)
