# Supplementary figures and images for: NF-κB Inducing Kinase, a Central Signaling Component of the Non-Canonical Pathway of NF-κB, Contributes to Ovarian Cancer Progression
Source: PLoS One. 2014 Feb 12;9(2):e88347. doi: 10.1371/journal.pone.0088347 (PMC3922808; doi:10.1371/journal.pone.0088347)

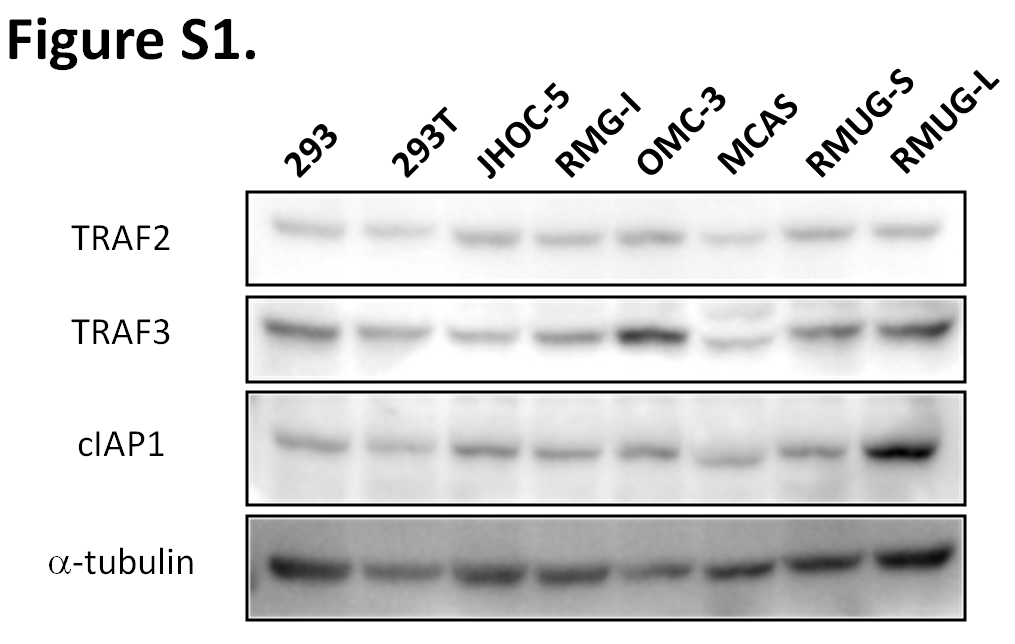

Supplement: Figure S1 — Thirty micrograms of cytoplasmic extracts were subjected to SDS-PAGE followed by immunoblotting with the anti-TRAF2, anti-TRAF3 or anti-cIAP1 antibodies. (TIF) [file pone.0088347.s001.tif]

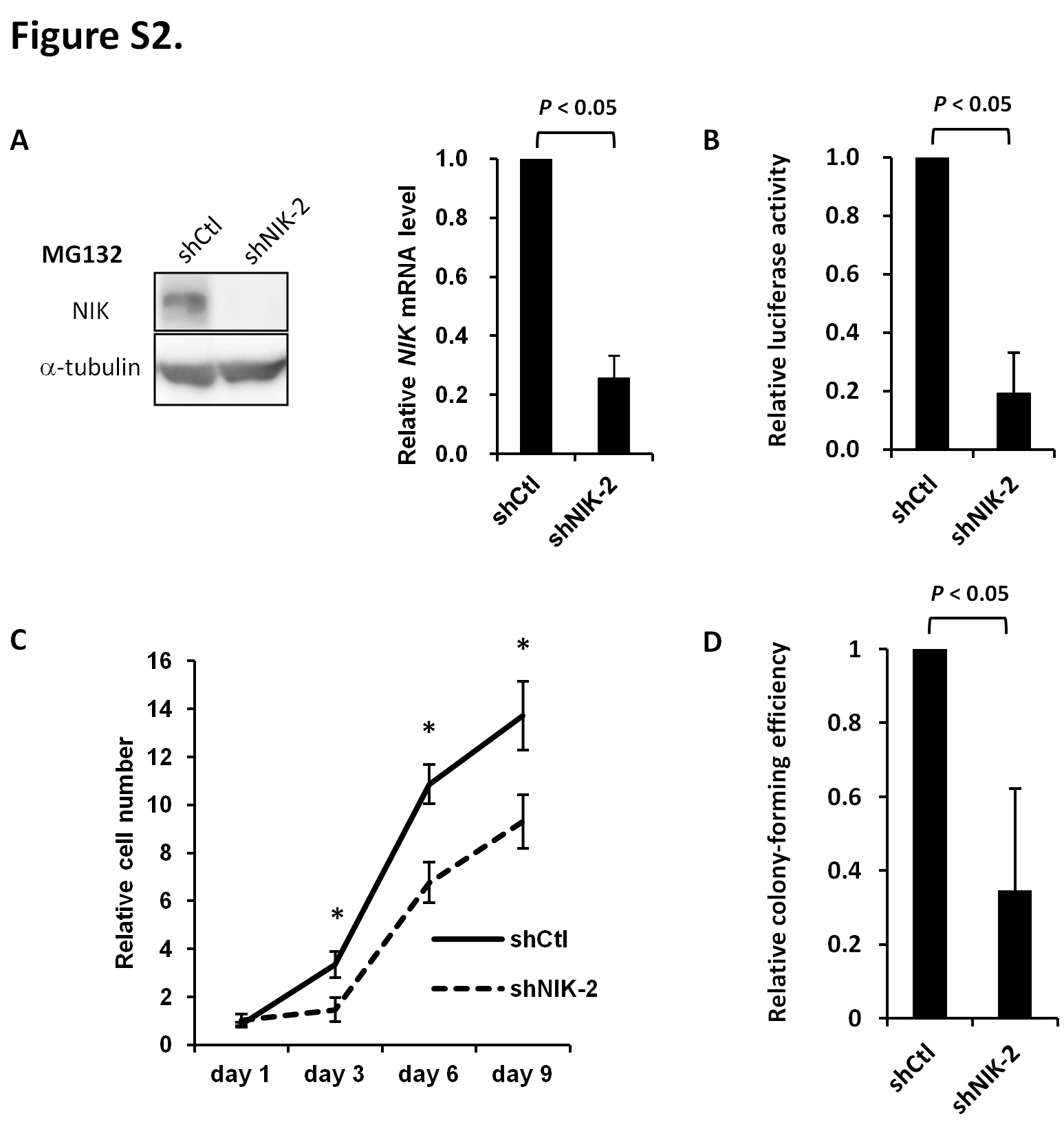

Supplement: Figure S2 — (A) RMG-I cells were infected with lentiviral vectors capable of expressing shRNA targeting NIK (shNIK-2) or GFP (shCtl) followed by selection with puromycin (4 µg/mL) for 72 hours. These cells were treated with 20 µM of MG132 for 6 hours and cytoplasmic extracts (30 µg) were subjected to SDS-PAGE and immunoblottings using anti-NIK or anti-α-tubulin antibodies. Expression of NIK mRNA was analyzed by real-time RT-PCR using total RNAs extracted these cells. (B) RMG-I cells transduced with lentiviruses carrying an NF-κB-dependent Firefly luciferase expression cassette and an EF-1α promoter-dependent Renilla luciferase expression cassette were infected with lentivirus vectors capable of expressing shRNA targeting NIK (shNIK-2) or GFP (Ctli). These cells were selected with puromycin (4 µg/mL) for 72 hours and subjected to the dual luciferase assay, in which Firefly luciferase activity was normalized by Renilla luciferase activity. Relative luciferase activities are expressed as light unit compared to the control (shCtl). (C) proliferation of NIK-depleted RMG-I cells in panel A was assessed by trypan blue exclusion test. Relative cell numbers is expressed as fold change compared to the number of cells plated. Single asterisks denote significant difference (P<0.05) between cells expressing shNIK-2 or shCtl. (D) NIK-depleted RMG-I cells in panel A were cultured in soft agar medium for three weeks and then cell colonies larger than 60 µm in diameter were counted under more than 20 microscopic fields at 40× magnification. Relative colony-forming efficiency is expressed as fold change compared to the control (shCtl). (TIF) [file pone.0088347.s002.tif]

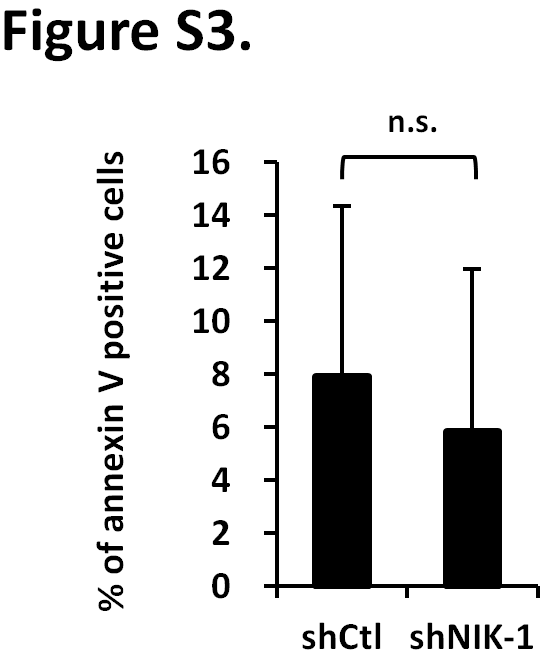

Supplement: Figure S3 — NIK-depleted RMG-I cells shown in Figure 5B were stained with FITC-conjugated Annexin-V and analyzed by flow cytometry. (TIF) [file pone.0088347.s003.tif]
