# Supplementary material for: NF-κB Inducing Kinase, a Central Signaling Component of the Non-Canonical Pathway of NF-κB, Contributes to Ovarian Cancer Progression
Source: PLoS One. 2014 Feb 12;9(2):e88347. doi: 10.1371/journal.pone.0088347 (PMC3922808; doi:10.1371/journal.pone.0088347)
Supplement: Methods S1 — Apoptosis assay. (DOC) [file pone.0088347.s004.doc]

**Supporting Information methods**

Apoptosis assay

Apoptotic cells were detected by staining with FITC-conjugated Annexin-V (BD Biosciences, 51-65874X) and samples were acquired with FACS Calibur system (BD Biosciences). The obtained data were analyzed by CellQuest software (BD Biosciences).
